# The association between self-esteem and aesthetic component of smile among adolescents

**DOI:** 10.1186/s40510-023-00508-w

**Published:** 2024-03-04

**Authors:** Lidia Gavic, Mihaela Budimir, Antonija Tadin

**Affiliations:** Study of Dental Medicine, School of Medicine, University of Split, Soltanska 2, 21000 Split, Croatia

**Keywords:** Adolescents, Aesthetic component, IOTN index, Self-esteem

## Abstract

**Objectives:**

Self-esteem plays a crucial role during adolescence in a shaping of an individual’s overall well-being and confidence. The aim of this study was to explore the relationship between the self-esteem in adolescents and their smile aesthetics, as well as to assess the alignment of opinions on this matter between adolescents and dentists.

**Methods:**

Sample included 413 students in Split-Dalmatia County, aged 13 to 18 (60% females). Data on demographic issues, orthodontic history, and desire for orthodontic treatment were collected by the self-administrated questionnaire. Coopersmith's Self-Esteem Inventory was also used. Smile aesthetics was assessed by each participant and dentist independently using an Aesthetic Component of the Index of Orthodontic Treatment Need (IOTN AC).

**Results:**

Self-esteem was higher in adolescents who rated their smile aesthetics equally to the dentist than in those who considered their aesthetics to be worse than the dentist (18.5 vs. 16; *P* = 0.011). The multiple linear regression revealed that the self-esteem of adolescents was positively related to undergone previous orthodontic treatment (*β* = 1.286, *P* = 0.020) while negatively related to the female gender (*β* = −2.531, *P* ≤ 0.001) and IOTN AC assessed by dentist (*β* = −0.356, *P* = 0.015). It was not related to educational level or desire for orthodontic treatment.

**Conclusion:**

The self-esteem in adolescence is influenced the most by gender, but the orthodontic treatment and better smile aesthetics might also contribute.

## Introduction

Self-esteem refers to the degree to which a person values, appreciates, approves of, and likes themselves [1]. It develops as a result of past experiences, influenced both by our own perceptions and the reactions we receive from our environment [2]. High self-esteem has been shown to be a predictor of success and well-being in various areas of life, including relationships, work, and health.

Adolescence represents a period of physical, psychological, and social changes but also intense social pressures, especially bringing a increased emphasis on physical attractiveness [3]. During this critical development stage, self-esteem plays a crucial role in shaping an individual’s overall well-being and confidence. Previous studies have shown that one-third of adolescents struggle with low self-esteem, especially in their early teenage years [4, 5]. The enhancement of one's physical appearance is positively correlated with their attitude, personality, and self-esteem [2]. Among various factors influencing physical attractiveness, dental aesthetics has a significant impact [6]. As a result, there is a prevalent inclination towards enhanced smile aesthetics in modern society. Patients actively pursue treatment options to achieve improved dentofacial aesthetics and undergo a positive transformation in their smiles [7–9]. Smile aesthetics are important not only in facial attractiveness but also in the perception of one's psychological traits [9]. Malocclusion refers to deviations from the normal skeletal and dental characteristics [10]. It is highly prevalent among children and young individuals [11]. While malocclusions typically do not pose a threat to oral function, they can negatively impact dentofacial aesthetics. This, in turn, can affect self-confidence, emotional development, and social integration. Consequently, many patients seek orthodontic treatment primarily for aesthetic purposes. The perceived attractiveness of one's face and smile plays a crucial role in the decision to undergo the orthodontic treatment. It is widely believed that such treatment can significantly influence the patient's physical appearance, interpersonal relationships, and psychological well-being [12]. In addition, studies have shown that dentofacial deviations and irregularities can lead to an imbalance in facial and dental aesthetics, consequently impacting self-confidence negatively [13].

Significant differences exist between the orthodontists’ and patients’ perceptions regarding the need for orthodontic treatment [10]. It is not uncommon for patients with a significant need for orthodontic therapy to express no aesthetic concerns, while others with ideal aesthetics may express unrealistic concerns. Furthermore, the desire for treatment does not always align with the actual clinical need [14]. To address this, the Index of Orthodontic Treatment Need (IOTN) was developed by Shaw and Brook in 1989. This index is one of the most widely used priority measures for identifying potential candidates who would benefit from reliable and standardized orthodontic treatment [15].

The Index of Orthodontic Treatment Need (IOTN) consists of two main components: the dental health component (DHC) and the aesthetic component (AC). The AC specifically focuses on evaluating and ranking the aesthetic aspects of an individual's teeth, considering various dental factors such as tooth alignment, spacing, crowding, and irregularities. By assessing these aesthetic elements, the AC provides valuable information to determine the level of aesthetic improvement required and guide orthodontic treatment planning [15].

The aim of this study was to examine the potential association between self-esteem in adolescents and their assessment of the IOTN AC of their smile, and the relationship between participants' self-esteem and the alignment of their self-assessed IOTN AC scores with those assessed by dentists. Furthermore, the study aimed to explore whether differences exist in self-esteem and the evaluation of the aesthetic component of smiles based on factors such as gender, age, and previous orthodontic treatment. To the best of our knowledge, this is the first study of its kind not only in the Republic of Croatia but in the Southeastern European region. The hypothesis of this study was that there is no relationship between adolescents' self-esteem and the evaluation of the aesthetic component of their smiles.

## Materials and methods

This research was approved by the Ethics Committee of the Faculty of Medicine of the University of Split (Class: 003-08/22-03/O0O3 Reg. No.:2 181-198-03-O4-22-OOL7). It was conducted through survey questionnaires handed to adolescents aged 13 to 18, in primary and secondary schools of the Split-Dalmatia County during 2022. Prior to commencing the study, the purpose and the methodology of the research were presented individually to each principal of the participating school and their professional services. Upon receiving their approval, the class teachers forwarded the informed consents to the parents of children planned to participate in the research. The children who participated in the research were also aware of the purpose of the research, and each respondent could withdraw from the participation at any time and for any reason. The questionnaire was completely anonymous and the adolescents filled it in independently.

In the Split-Dalmatia County, an average of 4 250 children are born annually, which makes a total of 21 250 children in the five examined generations. With a 5% margin of error, a 90% confidence interval, and a 50% response distribution, the minimum required sample size was 378.

The first part of the questionnaire contained questions about the general and demographic data of the respondents (age, gender, grade). Furthermore, the respondents were asked questions about previous orthodontic therapy or their desire for orthodontic treatment, to which they answered with yes and no.

In the second part, the respondents were presented with a photograph of the aesthetic component of the IOTN index. The respondents had to classify their smile according to one of the photographs. The aesthetic component consists of ten intraoral photographs that are classified according to aesthetic expression. They are rated from 1 to 10, with 1 being the best and 10 being the worst appearance. The orthodontist then would compare the appearance of the patient's teeth with photographs and classify them into one of ten grades. The comparison did not look for morphological similarities in the position of the teeth, but rather the aesthetic impression that the teeth create. This component classifies patients into three levels of need for the orthodontic therapy: the first level (Grade 1, 2, 3, 4) means that there is no need for the therapy, the second level (Grade 5, 6, 7) means that there is a moderate need, and the third level (Grade 8, 9, 10) means that there is a great need for therapy [15].

The third part of the questionnaire contained Coopersmith's self-esteem questionnaire, which is often used in scientific research on children and adolescents, and which the respondents filled out at their discretion and independently. The questionnaire consisted of 25 statements to which respondents had to choose one of the offered answers, true or false. The total score is the sum of all positive responses. The higher the score, the higher the respondent's self-esteem. The questionnaire was translated and adapted into Croatian by Lackovic Grgin [16].

At the end, when collecting the questionnaires, each respondent was asked to smile and the degree of the aesthetic component of the smile was professionally determined by the doctor of dental medicine according to the aesthetic component of the IOTN index.

### Statistical analysis

All correctly completed questionnaires were entered into Microsoft Excel 2007 (Microsoft Corporation, Redmond, Washington, USA) software. After the completion of the research, the data were subjected to statistical analysis using the SPSS software package (IBM Corp., Armonk, New York, USA). Descriptive analysis was conducted to calculate the frequency and percentage of categorical data, while quantitative data were presented as mean value, standard deviation, median, minimum, and maximum values. The distribution of responses within the self-esteem test was assessed using the Kolmogorov–Smirnov test. Statistical comparisons were performed using the Wilcoxon signed rank test and the Kruskal–Wallis test. Categorical variables were compared using the chi-square test. The results were presented in tables or figures. The significance level was set at *P* < 0.05.

## Results

**Fig. 1:**
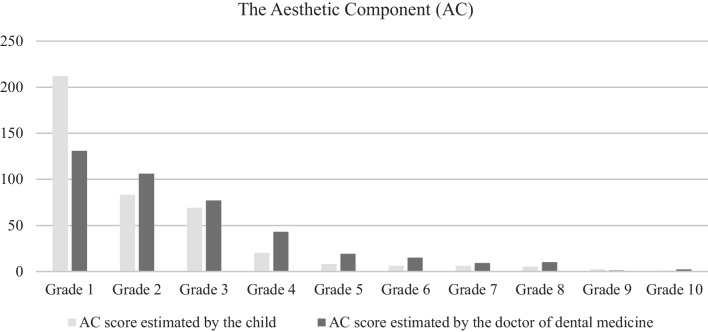
The assessment of smile aesthetics by the participant himself and by the doctor of dental medicine

In this study, 413 children participated, aged 13 to 18 (mean age 15.28 ± 1.61) out of which 60.29% were girls and 39.71% were boys. The most children (*N* = 212) graded their smile aesthetics with the Grade 1 (51.33%). Also, the doctor of dental medicine scored the most of the children’s smiles (*N* = 131) with the Grade 1 (31.72%) (Fig. 1). In the self-esteem questionnaire, participants achieved scores ranging from 2 to 25, with a mean value of 16.56 ± 5.19, median of 18 and interquartile range 8.

The Kolmogorov–Smirnov test showed that the variables were not normally distributed (*P* < 0.001).

Depending on the need for orthodontic treatment (no need, moderate, and great need according to their perceived smile aesthetics), there was a significant difference in the desire for orthodontic treatment among subjects according to their perceived smile aesthetics (*χ*^2^ = 9.017, *df* = 2, *P* = 0.011). Also, there was a significant difference in the desire for orthodontic treatment among subjects according to which group of need for orthodontic treatment doctors perceived their smile aesthetics (*χ*^2^ = 22.873, *df* = 2, *P* ≤ 0.001). The results of Z test with Bonferroni correction are presented in Table 1. More children without a need for orthodontic treatment lack a desire for it than expected, while more children with moderate or great needs express a desire for treatment than expected, whether perceived by themselves or by doctors. Furthermore, there was no difference in the desire for orthodontic treatment based on gender of the participants (*χ*^2^ = 2.153, *df* = 1, *P* = 0.142).

**Table 1:** Desire for orthodontic treatment among subjects depending on the need for orthodontic treatment (no need, moderate and great need) perceived by themselves or by doctors

	No need		Moderate need		Great need		
	Count	Expected count	Count	Expected count	Count	Expected count	
IOTN AC perceived by participants							
Desire for orthodontic treatment							
No	256	248.9	7	12.9	4	5.2	*χ*^2^ = 9.017, *df* = 2, *P* = 0.011
Yes	129	136.1	13	7.1	4	2.8	
IOTN AC perceived by doctors							
No	245	230.8	18	27.8	4	8.4	*χ*^2^ = 22.873, *df* = 2, *P* ≤ 0.001
Desire for orthodontic treatment							
Yes	112	126.2	25	152	9	4.6	

**Fig. 2:**
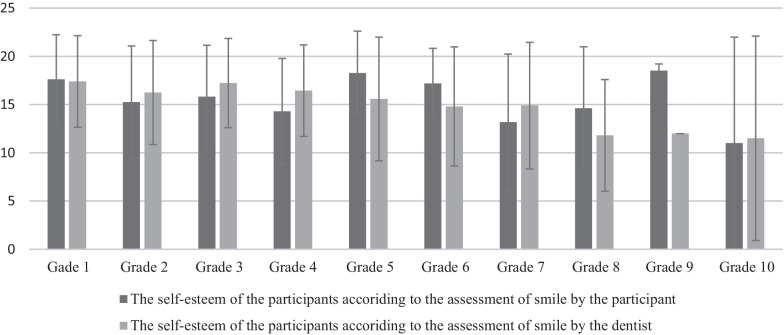
The self-esteem of the participants according to the assessment of smile by the participant and the dentist

Chi-square test and *Z* test intergroup comparison,

Table 2 presents the matchings between the assessment of smile aesthetics by the participants and the dentist, and self-esteem of the participants. The intraclass correlation coefficient, Cronbach's α, for average measures was 0.709 (*P* ≤ 0.001) which indicate acceptable consistency between the evaluation of the aesthetic component of the smile by the child and the doctor of dental medicine. Participants who rated their AC the same as the dentists had the highest self-esteem, while the participants who rated their AC lower than the dentist had the lowest self-esteem. The Kruskal–Wallis test showed a statistically significant difference in self-esteem concerning the evaluation of the AC (*P* = 0.020). In a pairwise comparison, a significant difference was found between the self-esteem of the subjects who rated their aesthetics the same as the dentists and the subjects who rated their smile aesthetics lower than the dentist (*P* = 0.011, adjusted by Bonferroni correction *P* = 0.032). Figure 2 shows the self-esteem of the respondents regarding the aesthetics of the smile. It is evident that children with better smile aesthetics also have higher self-esteem. When comparing self-esteem and self-assessment of smile aesthetics, a statistically significant difference was observed using the Kruskal–Wallis test (*P* = 0.007). Pairwise analysis showed a significant difference between children who graded the aesthetics of their smile as the Grade 1 and those who graded the aesthetics of their smile as the Grade 2 (*P* ≤ 0.001).

**Table 2:** The self-esteem of the participants according to the matching between the assessment of smile aesthetics by the participants and the dentist

	*N* (%)	Self-esteem of the participants M (IQR)
The participant and the dentist assessed the aesthetics of the smile equally	178 (43.10%)	18.5 (6)^a^
The participant that the aesthetic of his smile was better than the doctor of dental medicine estimated	177 (42.86%)	17 (7)
The participant estimated that the aesthetic of his smile was worse than the doctor of dental medicine estimated	58 (14.4%)	16 (7)^a^
		*P* = 0.020

*M* median, *IQR* interquartile range the Kruskal–Wallis test

^a^*P* = 0.011, adjusted by Bonferroni correction *P* = 0.032

Table 3 displays the self-esteem scores of the respondents in relation to orthodontic therapy and gender. The Mann–Whitney test revealed a statistically significant difference in self-esteem between boys and girls (*P* ≤ 0.001). Furthermore, the Mann–Whitney test indicated a significant difference in self-esteem between subjects who had received orthodontic treatment and those who had not (*P* = 0.002), as well as between subjects with different desire for orthodontic treatment (*P* = 0.006). However, there was no significant difference in self-esteem between subjects currently undergoing orthodontic therapy and those who were not (*P* = 0.113).

**Table 3:** Self-esteem of the subjects regarding gender and orthodontic treatment

		*N* (%)	M (IQR)	
Gender	Female	249	16 (5)	*P* ≤ 0.001
	Male	164	19 (8)	
Have you ever had orthodontic treatment?	No	247	16 (8)	*P* = 0.002
	Yes	166	19 (6)	
Do you currently have orthodontic treatment?	No	320	17 (7,75)	*P* = 0.113
	Yes	93	19 (7)	
Would you like to have orthodontic treatment?	No	267	18 (7)	*P* = 0.006
	Yes	146	16 (7)	

Mann–Whitney *U* test. *P* ≤ 0.05, *M* median, *IQR* interquartile range

The results of the linear regression analysis on the dependence of self-esteem on the predictors are shown in Table 4 (*R*^2^ = 0.104, *P* ≤ 0.001, for model). The self-esteem score correlates positively with previously undergone orthodontic treatment (*β* = 1.286, *P* = 0.020) and negatively with the female gender (β = −2.531, *P* ≤ 0.001) and IOTN AC index assessed by dentist (β = −0.356, *P* = 0.015).

**Table 4:** The results of the linear regression analysis on the dependence of self-esteem on the predictors variables

	Unstandardized coefficients		Standardized coefficients	*t*	*P*	Correlations		
	*B*	SE	Beta			Zero-order	Partial	Part
Desire for orthodontic treatment	−0.574	0.539	−0.053	−1.066	0.287	−0.130	0−.053	−0.050
Gender	−2.512	0.506	−0.237	−4.963	0.000	−0.215	−0.239	−0.233
previous orthodontic treatment	1.363	0.546	0.129	2.497	0.013	0.154	0.123	0.117
Education level	−0.075	0.104	−0.037	−0.721	0.471	−0.092	−0.036	−0.034
IOTN AC index assessed by dentist	−0.366	0.145	−0.129	−2.526	0.012	−0.180	−0.124	−0.119

## Discussion

The results obtained in this study revealed that the score individuals achieved on self-esteem questionnaire is associated with IOTN index. These findings are in accordance with other studies whose findings led to the conclusion that an improvement in self-esteem is among the most noteworthy outcomes of orthodontic treatment [6] and that enhanced physical attractiveness resulting from orthodontic treatment positively impacts self-esteem [17]. However, some studies did not provide evidence that there is a connection between orthodontic treatment and changes in self-esteem during adolescence [18].

In this study, a statistically significant difference was observed in the desire for orthodontic treatment among respondents when considering their year at school. However, a study conducted in Brazil did not find a statistically significant relationship in terms of the desire for orthodontic treatment among adolescents. Interestingly, in the mentioned study, as many as 78% of adolescents expressed a desire for orthodontic treatment [19]. In contrast, in our study, only 22% of adolescents expressed a desire for orthodontic treatment. However, some studies highlighted that gender was one of the significant factors influencing the desire for orthodontic treatment. Specifically, females expressed a significantly higher desire for orthodontic treatment in many studies [20, 21]. Contrary, in this study no difference was found in the desire for orthodontic treatment based on gender [19].

In our study, a significant correlation (*α* = 0.709, *P* ≤ 0.001) was found between the child's and the dentist's assessments of smile aesthetics. Similar results were obtained in the study conducted in Jordan [22]. Additionally, in the aforementioned study, the majority of respondents (91%) rated the aesthetics of their smile as aesthetically acceptable and placed it within the first four levels of the aesthetic component [22]. These findings are consistent with our own, where 93% of the respondents had similar ratings. It was observed that children tended to be less critical in their assessment of aesthetics compared to the dentists. In 42% of the cases, children rated their smile aesthetics better than the dentists did, while only 14% of cases involved children rating their aesthetics worse than the dentists’ assessment. Our study's findings are consistent with the study conducted between Jordanian adolescents where respondents were less critical of smile aesthetics compared to professionals [17]. It is important to emphasize that participants who rated the aesthetics of their smiles lower than the doctors also had lower scores on the self-esteem test. Those findings align with the results of previous studies, which have highlighted that individuals with lower self-esteem tend to have a distorted self-image of their appearance [23].

In our study, boys demonstrated significantly higher self-esteem than girls, which is in line with numerous studies, where girls exhibited lower self-esteem than boys [24, 25]. Moreover, several studies strongly suggest that the dissatisfaction with one’s body is a significant predictor of lower self-esteem in adolescent girls [23, 26]. Therefore, we can conclude that smile aesthetics can indeed play a significant role in the self-esteem of adolescents.

The results of our study indicate a significant association between self-esteem and the evaluation of the aesthetic component of one's smile. Adolescents with lower self-esteem tended to rate the aesthetic component of their smile lower, while those with higher self-esteem rated their smile aesthetics higher. These findings align with the research conducted by Badran et al., which also demonstrated a significant relationship between these two variables [17]. Moreover, adolescents who graded their smile aesthetics as Grade 1 had a significant difference in self-esteem compared to those who graded their smile aesthetics as Grade 2. Since we are considering "perfect smile aesthetics" (Grade 1) and "almost perfect smile aesthetics" (Grade 2), the decision of the child to which group they belong is closely linked to their self-esteem.

The results of our study indicate that previous orthodontic treatment have a positive with self-esteem. These findings are consistent with other studies that have shown that orthodontic treatment during adolescence can have a positive effect on the self-esteem [27, 28].

Furthermore, the results of the regression analysis have indicated that gender plays a crucial role in self-esteem levels. However, other examined predictors have also proven to be significant, such as the aesthetics of the smile and prior orthodontic treatment. Therefore, we can conclude that smile aesthetics can indeed play a significant role in the self-esteem of adolescents.

In our study, respondents who expressed a desire for orthodontic treatment demonstrated significantly lower self-esteem compared to those who did not have this desire. Similar results were reported by Badran et al*.,* where respondents who expressed a desire for orthodontic treatment also exhibited lower self-esteem [17].

Furthermore, no statistically significant difference in self-esteem was found between adolescents who are currently undergoing orthodontic treatment and those who are not. These results are consistent with the findings of Jung's study, which suggested that fixed orthodontic treatment has no effect on self-esteem while treatment is ongoing [27].

This study examining the association between self-esteem and the aesthetics of a smile holds the potential to yield numerous benefits and enhance our understanding of this relationship. Insight into the impact of smile aesthetics on social interactions, well-being, social integration, and interpersonal relationships could assist dentists and other healthcare professionals deliver more comprehensive care, particularly to adolescents.

Based on the obtained results, orthodontic treatment might contribute to the improvement of adolescents' self-esteem. However, nevertheless, it is important to note that our study was cross-sectional and did not account for other factors contributing to self-esteem for each respondent, which represents a limitation of this study and is also the limitation of this study. Additionally, it remains unknown what the initial self-esteem levels were for adolescents who had undergone orthodontic treatment. Therefore, it is recommended to conduct more extensive longitudinal studies that compare the self-esteem of individuals before and after orthodontic treatment. Moreover, future studies should include participants from various age groups, not limited to adolescents.

In conclusion, there is an association between self-perception of smile aesthetics and self-esteem during adolescence. While a smile plays a role in influencing self-confidence and, consequently, self-esteem, it is just one component in a broader array of factors. Namely, self-esteem and smile aesthetics are mutually dependent, meaning that individuals with higher self-esteem tend to perceive their imperfections with less severity. Although self-esteem is a multidimensional construct and self-perception of smile aesthetics is not the sole determinant of self-esteem, understanding this relationship can aid in developing targeted interventions and strategies to support adolescents in building self-esteem and addressing dental aesthetic concerns.

## Data Availability

The data underlying this article will be shared on reasonable request to the corresponding author.
